# PARP12 (ARTD12) suppresses hepatocellular carcinoma metastasis through interacting with FHL2 and regulating its stability

**DOI:** 10.1038/s41419-018-0906-1

**Published:** 2018-08-28

**Authors:** Changjuan Shao, Yangyang Qiu, Juan Liu, Huan Feng, Suqin Shen, Hexige Saiyin, Wenbo Yu, Youheng Wei, Long Yu, Wei Su, Jiaxue Wu

**Affiliations:** The State Key Laboratory of Genetic Engineering, School of Life Science, Zhongshan Hospital, Fudan University, Shanghai, China

## Abstract

PARP12 is a mono-ADP-ribosyltransferase, but its function remains largely unknown. Here, we identified four-and-a-half LIM-only protein 2 (FHL2) as a functional partner of PARP12 through protein affinity purification. Although PARP12 did not mono-ADP-ribosylate FHL2 in vitro and in vivo, PARP12 deficiency decreased the protein level of FHL2 by promoting its ubiquitination and increased the expression level of transforming growth factor beta1 (TGF-β1), which is independent of PARP12 enzymatic activity. We also provided evidence that PARP12 deficiency increased the migration and invasion of hepatocellular carcinoma (HCC) cells and promoted HCC metastasis in vivo by regulating the epithelial–mesenchymal transition process. These results indicated that PARP12 is a tumor suppressor that plays an important role in HCC metastasis through the regulation of FHL2 stability and TGF-β1 expression.

## Introduction

ADP-ribosylation is an evolutionarily conserved post-translational modification that plays important roles in expanding the range of cellular functions, such as DNA repair and replication, chromatin remodeling, transcription, and telomere homeostasis^1,2^. ADP-ribosylation is mainly catalyzed by intracellular ADP-ribosyltransferases (ARTs), which use nicotinamide adenine dinucleotide (NAD^+^) to transfer ADP-ribose moieties to specific residues on target proteins, leading to mono-ADP-ribosylation (MARylation) or the formation of linear or branched chains of poly-ADP-ribose (PARylation)^1,2^. The functions of PARylation are relatively well characterized, and its inhibitors have been extensively investigated for the treatment of various cancer types, especially in ovarian cancer and breast cancer involving BRCA1/2 mutation^3,4^. In contrast to PARylation, the specific roles of MARylation are much less understood. MARylation is involved in transcriptional regulation, unfolded protein response, DNA repair, insulin secretion, immunity, and cancer development^5–7^. In mammals, at least 16 ADP-ribosyltransferases, including the cholera toxin-like ART family, the majority of the diphtheria toxin-like ART (ARTD) family, and some of the sirtuin family, catalyze MARylation^8^.

Poly(ADP-ribose) polymerase 12 (PARP12), also known as ARTD12, is a mono-ADP-ribosyltransferase. It was originally identified as a putative antiviral gene belonging to a large family of interferon-stimulated genes whose expression is often induced during viral infections^9,10^. PARP12 expression is also induced by bacterial superantigen-(SEB)-mediated toxic shock^11,12^. PARP12 contains five typical CCCH zinc fingers, two WWE domains, and a catalytic domain^11,13^. The zinc fingers of PARP12 are associated with viral and cytoplasmic RNAs^14^. PARP12 can translocate to cytoplasmic stress granules in response to stress, which is dependent on its WWE domain association with poly-ADP-ribose polymers catalyzed by PARP1^15^. PARP12 also inhibits cellular translation and virus replication by directly binding to the polysomes of Venezuelan equine encephalitis-infected cells^10,12^. However, the function of PARP12 in cancer development remains largely unknown.

In the present study, we found that PARP12 is associated with FHL2 and implicated in the regulation of its stability, thereby negatively regulating TGF-β1 expression and EMT processes. PARP12 deficiency promotes the migration and invasion of HCC cells and increases HCC metastasis in vivo. Our results indicated that PARP12 is a tumor suppressor and may be a novel therapeutic option for HCC treatment.

## Results

### PARP12 interacts with FHL2

**Fig. 1:**
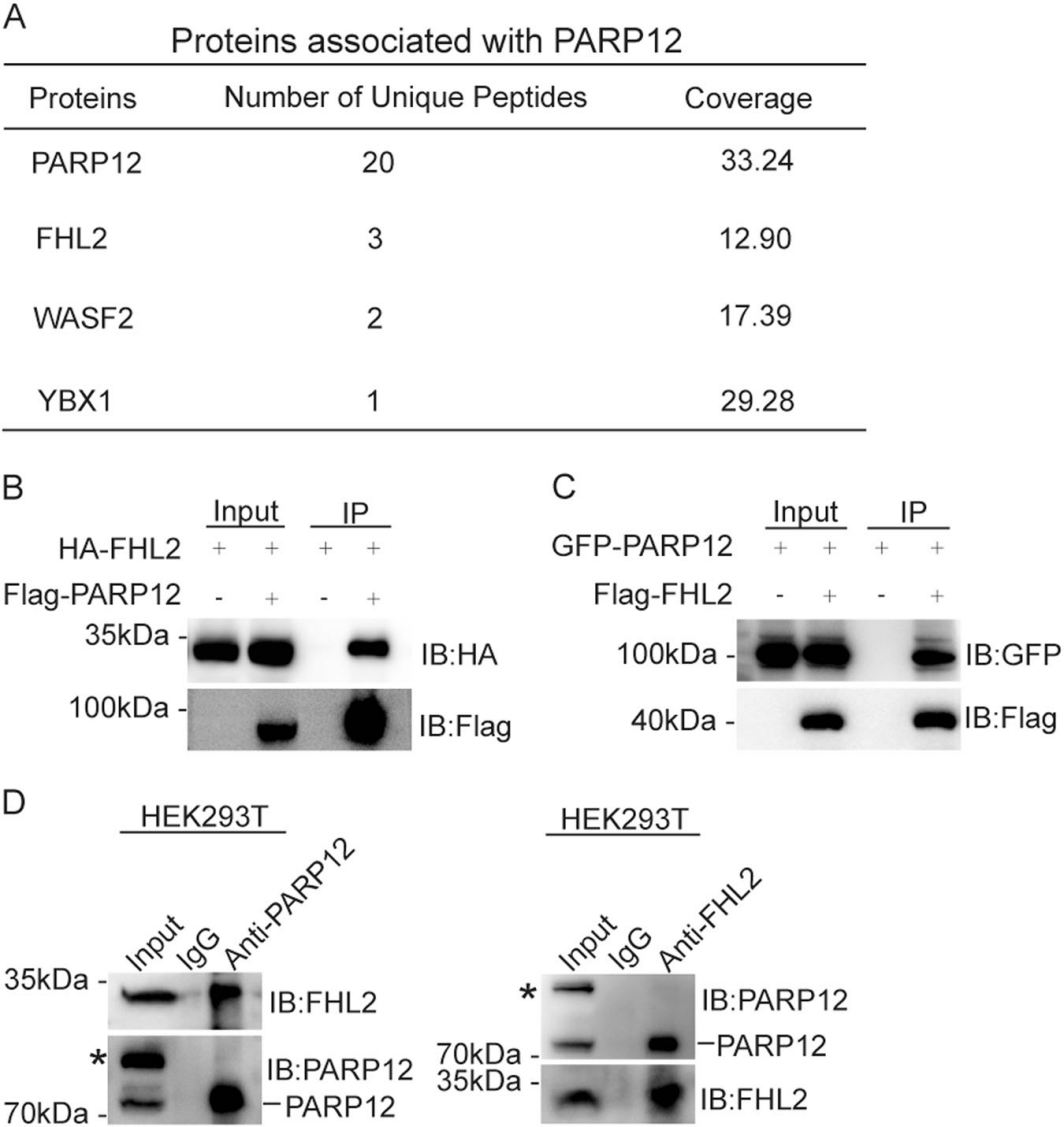
PARP12 interacts with FHL2. **a** FHL2 was identified to be a PARP12-associated protein by affinity purification. Proteins identified in the PARP12 affinity purification complexes are listed with the number of unique peptides found and the coverage according to mass spectrometry analysis. **b**, **c** HA-FHL2 and SFB-PARP12 or GFP-PARP12 and SFB-FHL2 were co-transfected into HEK293T cells and applied to immunoprecipitation (IP) followed by Western blot using the indicated antibodies. Whole-cell lysates were blotted and shown as input. **d** Endogenous PARP12 interacts with FHL2. Lysates from HEK293T cells were subjected to IP and Western blot with the indicated antibodies. An irrelevant IgG was used as the negative control. *: non-specific bands

To identify the functional partners of PARP12, we generated HEK293T cells that stably expressed streptavidin-Flag-S protein (SFB)-tagged PARP12 and conducted tandem affinity purification. Mass spectrometry analysis revealed that FHL2, a LIM-only protein that belongs to the four-and-a-half LIM-only protein family, was present in the PARP12 affinity purification complex (Fig. 1a). Then, we performed exogenous and endogenous reciprocal immunoprecipitation (IP) assays to validate the interaction between PARP12 and FHL2. As shown in Fig. 1b, c, the exogenously expressed HA-tagged FHL2 interacted with SFB-tagged PARP12, and GFP-tagged PARP12 interacted with SFB-tagged FHL2. Next, we examined the interaction of endogenous PARP12 and FHL2 in HEK293T, QGY-7703, and Huh7 cells by using anti-PARP12 and anti-FHL2 antibodies to perform endogenous Co-IP. As shown in Fig. 1d and Supplementary Figure 1, endogenous PARP12 and FHL2 formed a complex in all the examined cells. These results indicated that FHL2 was a partner of PARP12.

### FHL2 is not mono-ADP-ribosylated by PARP12

**Fig. 2:**
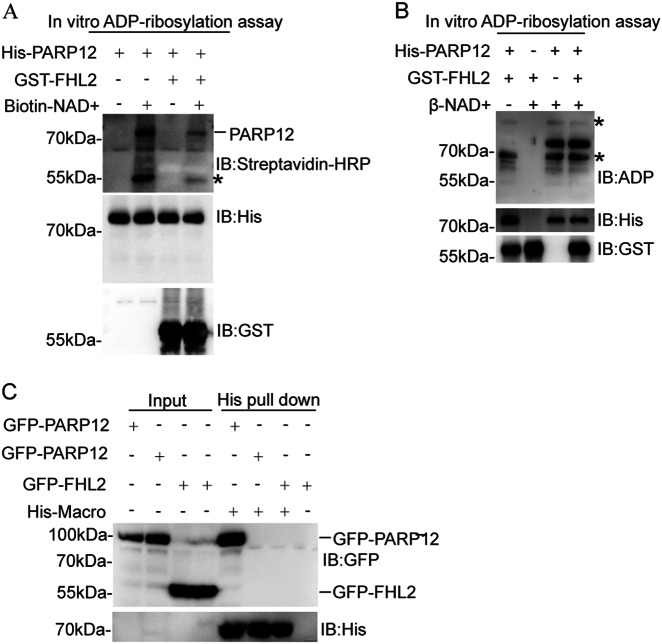
FHL2 is not modified by PARP12 in vitro and in vivo. **a**, **b** FHL2 is not modified by PARP12 as examined by an in vitro ADP-ribosylation assay. GST-FHL2 (1 µg) was incubated with His-PARP12 (0.5 µg) in the presence or absence of biotin-NAD^+^ or β-NAD^+^ (25 µM) at 30 °C for 30 min. The reactions were stopped by the addition of 2 × SDS loading buffer and boiled at 98 °C for 10 min. The samples were analyzed by Western blot using streptavidin-HRP (**a**) or anti-ADP-ribose (**b**) antibody. *: non-specific bands. **c** FHL2 is not mono-ADP-ribosylated in vivo. GFP-PARP12 WT, GFP-PARP12 MT (H564Y G565A), and GFP-FHL2 were transfected into HEK293T cells. Whole-cell lysates were subjected to pull-down assays using the His-tagged macro domains 1–3 of PARP14 coupled with Ni Sepharose. Proteins were separated by SDS-PAGE gel and analyzed by Western blot using the indicated antibodies. *: non-specific bands

Considering that FHL2 interacts with PARP12 and that PARP12 is a mono-ADP-ribosyltransferase, we proposed that FHL2 was likely mono-ADP-ribosylated by PARP12. To test this hypothesis, we expressed and purified His-tagged PARP12 and GST-tagged FHL2 from *Escherichia coli* and used these purified fusion proteins and biotinylated NAD^+^ to perform an in vitro mono-ADP-ribosylation assay. Western blot involving streptavidin-HRP revealed that His-PARP12 was mono-ADP-ribosylated by itself in the presence of biotinylated NAD^+^ (Fig. 2a). However, GST-FHL2 was not mono-ADP-ribosylated by His-PARP12 in the same reaction (Fig. 2a), suggesting that FHL2 was not the substrate of PARP12 in vitro. Anti-(ADP-ribose) antibody is specific to mono-ADP-ribose and can be used to detect mono-ADP-ribosylated polypeptides^16^. Hence, we performed an in vitro mono-ADP-ribosylation assay by using His-PARP12, GST-FHL2, and β-NAD^+^. After the reaction occurred, the samples were analyzed through Western blot by using the anti-(ADP-ribose) antibody. In Fig. 2b, His-PARP12, not GST-FHL2, was detected by anti-(ADP-ribose) antibody, further suggesting that FHL2 was not mono-ADP-ribosylated by PARP12 in vitro.

The macro domain of PARP14 is a “reader” module of mono-ADP-ribose and can pull down mono-ADP-ribosylated proteins from cells^17,18^. We expressed and purified the His-tagged macro domains 1–3 of PARP14 from *E. coli* and used these purified fusion proteins to pull down GFP-tagged PARP12 and FHL2 from the HEK293T cells. In Fig. 2c, wild-type GFP-PARP12, not enzyme-inactive mutant GFP-PARP12, was successfully pulled down by His-macro domains, indicating that GFP-PARP12 was mono-ADP-ribosylated in cells. However, GFP-FHL2 failed to be pulled down by His-macro domains, further suggesting that FHL2 could not be mono-ADP-ribosylated in vivo. Therefore, FHL2 was unlikely a substrate of PARP12.

### PARP12 regulates the stability of FHL2

**Fig. 3:**
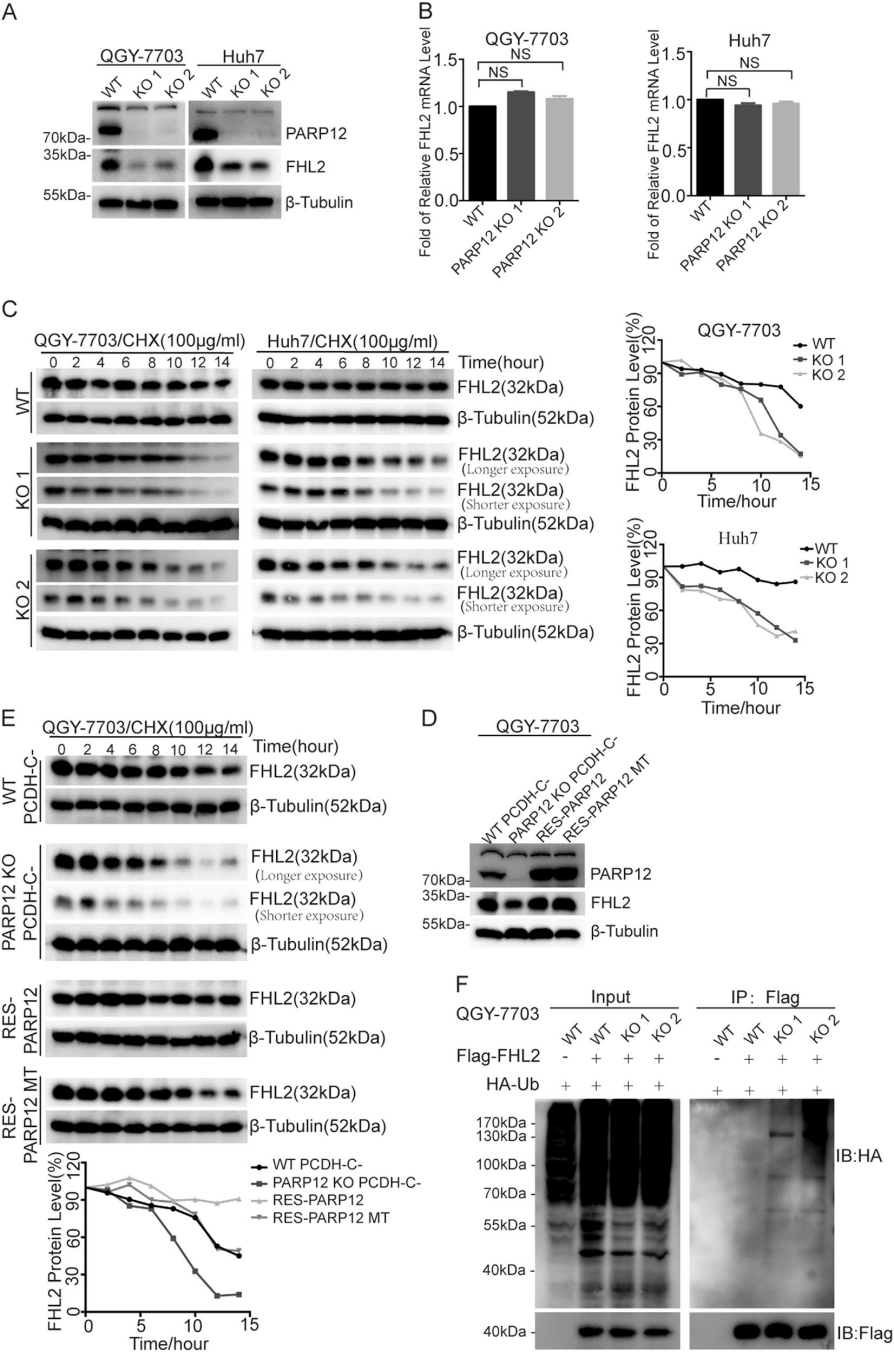
PARP12 regulates the stability of FHL2. **a** PARP12 deficiency decreased the protein level of FHL2. The protein levels of PARP12 and FHL2 in wild-type or PARP12-deficient QGY-7703 and Huh7 cells were examined by Western blot using the indicated antibodies. β-tubulin was used as a loading control. **b** PARP12 deficiency did not affect the mRNA level of FHL2. The mRNA level of FHL2 in PARP12 wild-type or -deficient cells were analyzed by RT-qPCR. “NS”: *P* > 0.05. **c** PARP12 regulates the protein stability of FHL2. Wild-type or PARP12-deficient QGY-7703 and Hun7 cells were treated with 100 µg/ml cycloheximide (CHX), collected at the indicated time points and immunoblotted with antibodies against FHL2 and β-tubulin. Quantification of FHL2 protein levels (normalized to β-tubulin) by ImageJ software is shown. **d** Wild-type and enzyme-inactive mutant PARP12 reconstitutions increased the protein level of FHL2 in PARP12-deficient cells. The protein levels of PARP12 and FHL2 in PARP12 wild-type, deficient, and reconstituted cells were examined by Western blot using the indicated antibodies, where β-tubulin was used as a loading control. **e** Wild-type and enzyme-inactive mutant PARP12 reconstitutions stabilized FHL2 in PARP12-deficient cells. Wild-type or PARP12-deficient and -reconstituted QGY-7703 cells were treated with 100 µg/ml CHX, collected at the indicated time points and immunoblotted with antibodies against FHL2 and β-tubulin. Quantification of FHL2 protein levels (normalized to β-tubulin) by ImageJ software is shown. **f** PARP12 deficiency promoted the ubiquitination of FHL2. Wild-type and PARP12-deficient QGY-7703 cells were co-transfected with SFB-FHL2 and HA-Ub, then pulled down by streptavidin agarose and analyzed by Western blot using the indicated antibodies

Next, we generated PARP12-deficient cell lines by using the CRISPR-Cas9 system to examine whether PARP12 regulates the function of FHL2. Considering that FHL2 plays important roles in HCC development^19,20^, we chose the QGY-7703 and Huh7 cell lines to generate PARP12-deficient cells. As shown in Supplementary Figure 2A, sgRNA targeting the exon 2 of PARP12 was designed, cloned into a CRISPR-Cas9 vector, and transfected into QGY-7703 and Huh7 cells. After the cells were selected, single colonies were screened through Western blot by using anti-PARP12 antibodies. Unlike wild-type cells, PARP12 proteins could not be detected through Western blot by using anti-PARP12 antibodies in PARP12-deficient QGY-7703 and Huh7 cells (Fig. 3a). DNA sequencing revealed that the genomic DNA region of PARP12 exon 2 in PARP12-deficient QGY-7703 and Huh7 cells contained the deletions or insertions of several base pairs, thereby leading to the frame shift of the open reading frame of PARP12 (Supplementary Figure 2B).

Western blot involving anti-FHL2 antibody demonstrated that the protein levels of FHL2 in PARP12-deficient QGY-7703 and Huh7 cells decreased compared with those of the wild-type cells (Fig. 3a). However, the mRNA levels of FHL2 did not significantly change in PARP12-deficient cells compared with those of the wild-type QGY-7703 and Huh7 cells (Fig. 3b). These results indicated that PARP12 might regulate the stability of FHL2 protein. We performed a cycloheximide (CHX) treatment assay to examine the half-life of FHL2 in PARP12 wild-type and deficient cells. In Fig. 3c, the half-life of FHL2 decreased in PARP12-deficient QGY-7703 and Huh7 cells compared with that of the wild-type cells after CHX treatment was administered. To further confirm this result, we reconstituted the wild-type PARP12 and the inactive mutant PARP12 into PARP12-deficient cells and examined the protein level of FHL2 through Western blot by using anti-FHL2 antibody. In Fig. 3d and Supplementary Figure 3A, the protein level of FHL2 increased in both wild-type and inactive mutant PAR12-reconstituted QGY-7703 and Huh7 cells compared with that of the PARP12-deficient cells. The CHX treatment experiments also indicated that both wild-type and inactive mutant PAR12 could rescue the half-life of FHL2 in PARP12-deficient cells (Fig. 3e and Supplementary Figure 3B). These results indicated that PARP12 interacted with FHL2 and regulated its stability, but these activities were independent of the enzyme activity of PARP12.

Protein stability is mainly mediated by protein ubiquitination^21^. PARP12 regulates FHL2 stability, suggesting that PARP12 might regulate the ubiquitination of FHL2. To test this hypothesis, we co-transfected SFB-FHL2 and HA-Ub into wild-type or PARP12-deficient cells and examined the ubiquitination level of SFB-FHL2 through IP followed by Western blot. In Fig. 3f, the ubiquitination level of SFB-FHL2 increased in PARP12-deficient cells compared with that of the wild-type cells, indicating that PARP12 deficiency might promote the ubiquitination of FHL2.

E3 ligase TRAF6 interacts with and ubiquitinates FHL2, thereby regulating its proteasome degradation^22^. Considering that PARP12 and TRAF6 interact with FHL2, we determined whether PARP12 and TRAF6 were competitively associated with FHL2. Flag-TRAF6 and HA-FHL2 were co-transfected into wild-type or PARP12-deficient QGY-7703 cells, and the interaction of Flag-TRAF6 and HA-FHL2 was examined through Co-IP and Western blot by using the indicated antibodies. In supplementary Figure 4, the interaction of TRAF6 and FHL2 was more enhanced in PARP12-deficient cells compared with that of the wild-type cells, suggesting that PARP12 suppressed the interaction of TRAF6 and FHL2 and negatively regulated the ubiquitination of FHL2 by TRAF6.

### PARP12 negatively regulates TGF-β1 expression through FHL2

**Fig. 4:**
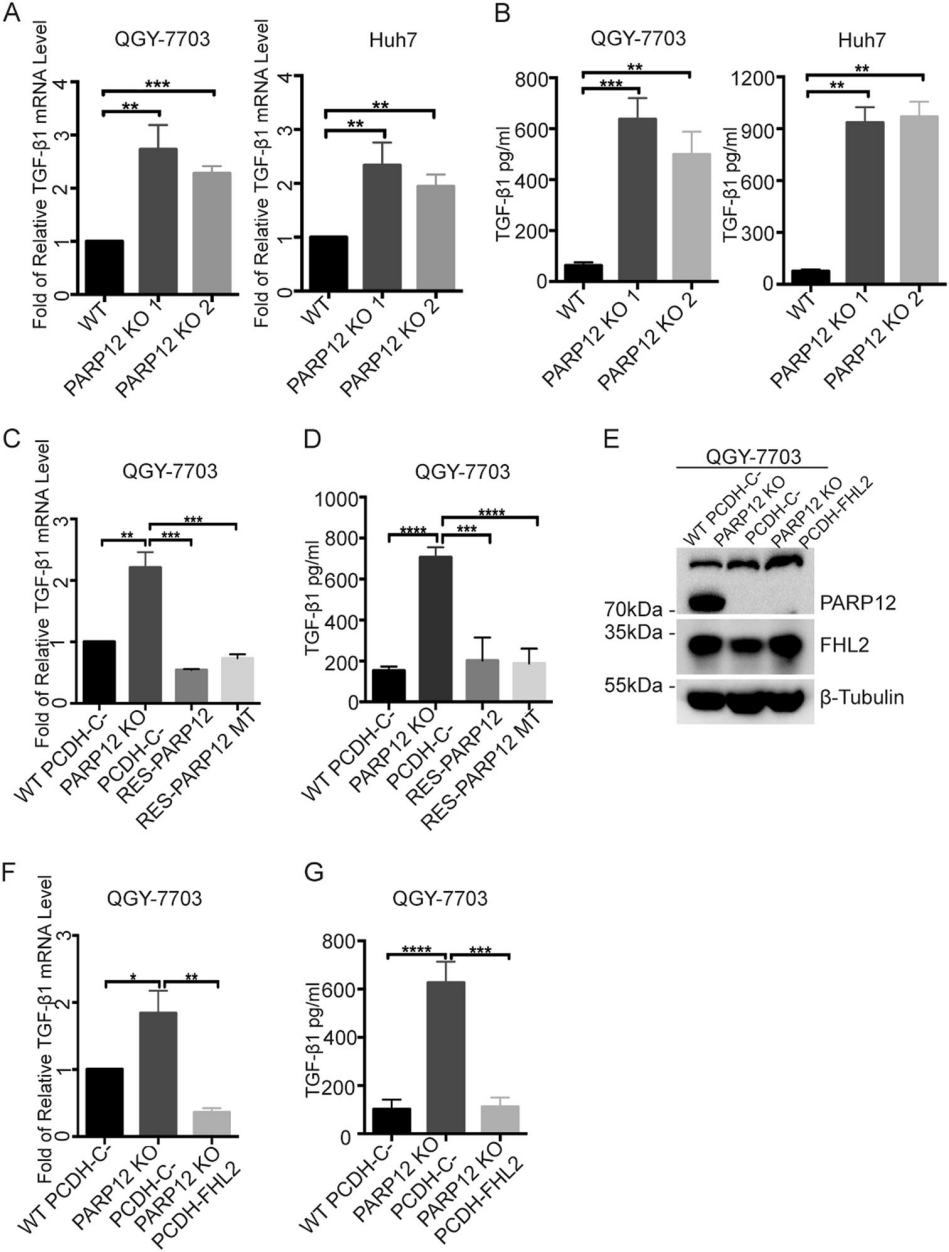
PARP12 negatively regulates TGF-β1 expression through FHL2. **a** PARP12 deficiency increased the transcription of TGF-β1. The mRNA levels of TGF-β1 in PARP12 wild-type or -deficient cells were analyzed by RT-qPCR. **P < 0.01, ****P < 0.0001. **b** PARP12 deficiency increased the TGF-β1 level in cell culture medium. The TGF-β1 levels in the cell culture mediums of PARP12 wild-type or -deficient cells were analyzed by ELISA. **P < 0.01, ***P < 0.001. **c** Wild-type and enzyme-inactive mutant PARP12-reconstitutions decreased the transcription of TGF-β1 in PARP12-deficient cells. The mRNA levels of TGF-β1 in PARP12 wild-type, -deficient, and -reconstituted cells were analyzed by RT-qPCR. **P < 0.01, ***P < 0.001, ****P < 0.0001. **d** Wild-type and enzyme-inactive mutant PARP12 reconstitutions decreased the TGF-β1 level in PARP12-deficient cell culture medium. The TGF-β1 levels in PARP12 wild-type, -deficient, and -reconstituted cell culture mediums were analyzed by ELISA. ***P < 0.001, ****P < 0.0001. **e** PARP12 regulated TGF-β1 expression dependent on FHL2. FHL2 was overexpressed in PARP12-deficient QGY-7703 cells, and the expression level was examined by Western blot using the indicated antibodies. **f**, **g** The mRNA levels of TGF-β1 and the secretory TGF-β1 levels in wild-type, PARP12-deficient, and FHL2-overexpressed PARP12-deficient cells were analyzed by RT-qPCR and ELISA, respectively. *P < 0.05, **P < 0.01, ***P < 0.001, ****P < 0.0001

FHL2 is a negative regulator of TGF-β1, not TGF-β2/3, in the liver^23,24^. We also found that FHL2 deficiency increased the transcription of TGF-β1 in HCC cell lines (data not shown), suggesting that FHL2 suppressed the TGF-β1 expression in HCC cells. Considering that PARP12 regulated the stability of FHL2, we raised the possibility that PARP12 regulated the expression of TGF-β1 in HCC cells. Hence, we examined the expression level of TGF-β1 in wild-type and PARP12-deficient cells through RT-qPCR. In Fig. 4a, the mRNA levels of TGF-β1 increased in PARP12-deficient QGY-7703 and Huh7 cells compared with those of the wild-type cells. TGF-β1 is a secretory cytokine. We examined the TGF-β1 level in the cell culture medium of wild-type and PARP12-deficient cells through ELISA. In Fig. 4b, the TGF-β1 level increased in the medium of PARP12-deficient cells compared with that of the wild-type cells. The mRNA levels of TGF-β1 and the protein level of TGF-β1 in the cell culture medium decreased when the wild-type or enzyme inactive mutant PARP12 was re-constituted into PARP12-deficient QGY-7703 and Huh7 cells (Fig. 4c, d and Supplementary Figure 5). These results indicated that PARP12 negatively regulated the TGF-β1 expression in HCC cells.

To examine whether the regulation of TGF-β1 expression by PARP12 was dependent on the protein level of FHL2, we overexpressed FHL2 in PARP12-deficient QGY-7703 cells to analyze the expression level of TGF-β1. As shown in Fig. 4e, the protein level of FHL2 in PARP12-deficient QGY-7703 cells was similar to that of the wild-type cells when FHL2 was overexpressed in PARP12-deficient cells. As expected, the mRNA and protein levels of TGF-β1 in the cell culture medium decreased when FHL2 was overexpressed in PARP12-deficient QGY-7703 cells (Fig. 4f, g), suggesting that the regulation of TGF-β1 expression by PARP12 was dependent on FHL2. These results indicated that PARP12 negatively regulated TGF-β1 expression via FHL2.

### PARP12 regulates the epithelial–mesenchymal transition (EMT) process

**Fig. 5:**
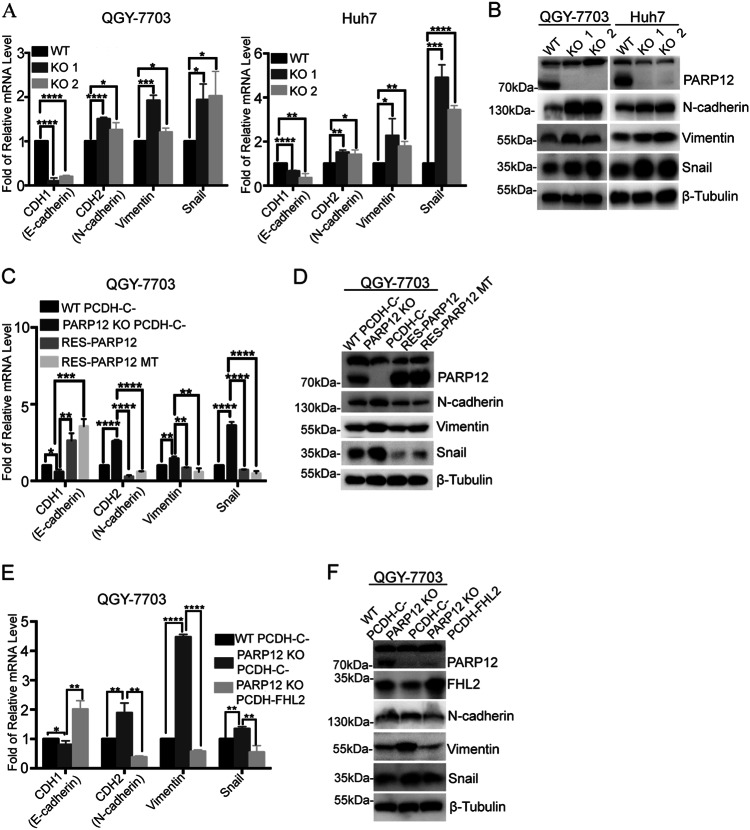
PARP12 regulates the EMT process. **a** PARP12 regulates the expression of EMT markers. The mRNA levels of CDH1, CDH2, Vimentin, and Snail1 were examined in PARP12 wild-type or -deficient QGY-7703 and Huh7 cells by RT-qPCR. *P < 0.05, **P < 0.01, ***P < 0.001, ****P < 0.0001. **b** The protein levels of N-Cadherin, Vimentin, and Snail1 in PARP12 wild-type or -deficient QGY-7703 and Huh7 cells were examined by Western blot using the indicated antibodies, where β-tubulin was used as a loading control. **c** The mRNA levels of CDH1, CDH2, Vimentin, and Snail1 were examined in PARP12 wild-type, -deficient, and -reconstituted QGY-7703 cells by RT-qPCR. *P < 0.05, **P < 0.01, ***P < 0.001, ****P < 0.0001. **d** The protein levels of N-Cadherin, Vimentin, and Snail1 in PARP12 wild-type, wild-deficient, and wild-reconstituted QGY-7703 cells were examined by Western blot using the indicated antibodies, where β-tubulin was used as a loading control. **e** The mRNA levels of CDH1, CDH2, Vimentin, and Snail1 were examined in PARP12 wild-type, PARP12-deficient, and FHL2-overexpressed PARP12-deficient QGY-7703 cells by RT-qPCR. *P < 0.05, **P < 0.01, ****P < 0.0001. **f** The protein levels of N-Cadherin, Vimentin, and Snail1 in PARP12 wild-type, PARP12-deficient, and FHL2-overexpressed PARP12-deficient QGY-7703 cells were examined by Western blot using the indicated antibodies, where β-tubulin was used as a loading control

TGF-β1 is a potent EMT driver that plays critical roles in the EMT process^25,26^. Considering that PARP12 negatively regulates TGF-β1 expression, we hypothesized that PARP12 might regulate EMT. To test this hypothesis, we analyzed the expression of EMT-associated markers in wild-type or PARP12-deficient cells through RT-qPCR and Western blot. As shown in Fig. 5a, the mRNA level of E-cadherin, an epithelial cell marker, decreased in PARP12-deficient cells compared with that of the wild-type cells. On the contrary, the mRNA and protein levels of mesenchymal markers, such as N-cadherin, Vimentin, and Snail1, increased in PARP12-deficient cells compared with those of the wild-type cells (Fig. 5a, b), suggesting that PARP12 deficiency promoted the EMT process. The expression level of E-cadherin increased in PARP12-deficient cells after wild-type or enzyme-inactive mutant PARP12 reconstitution occurred (Fig. 5c and Supplementary Figure 6A). The expression levels of N-cadherin, Vimentin, and Snail1 decreased in PARP12-deficient cells after wild-type or enzyme-inactive mutant PARP12 reconstitution was completed (Fig. 5c, d and Supplementary Figures 6A and 6B). The expression level of E-cadherin increased, whereas the expression levels of N-cadherin, Vimentin, and Snail1 decreased when FHL2 was overexpressed in PARP12-deficient QGY-7703 cells (Fig. 5e, f), suggesting that the regulation of EMT by PARP12 was dependent on FHL2. These results suggested that PARP12 regulated EMT through FHL2.

### PARP12 deficiency promotes cell migration and invasion

**Fig. 6:**
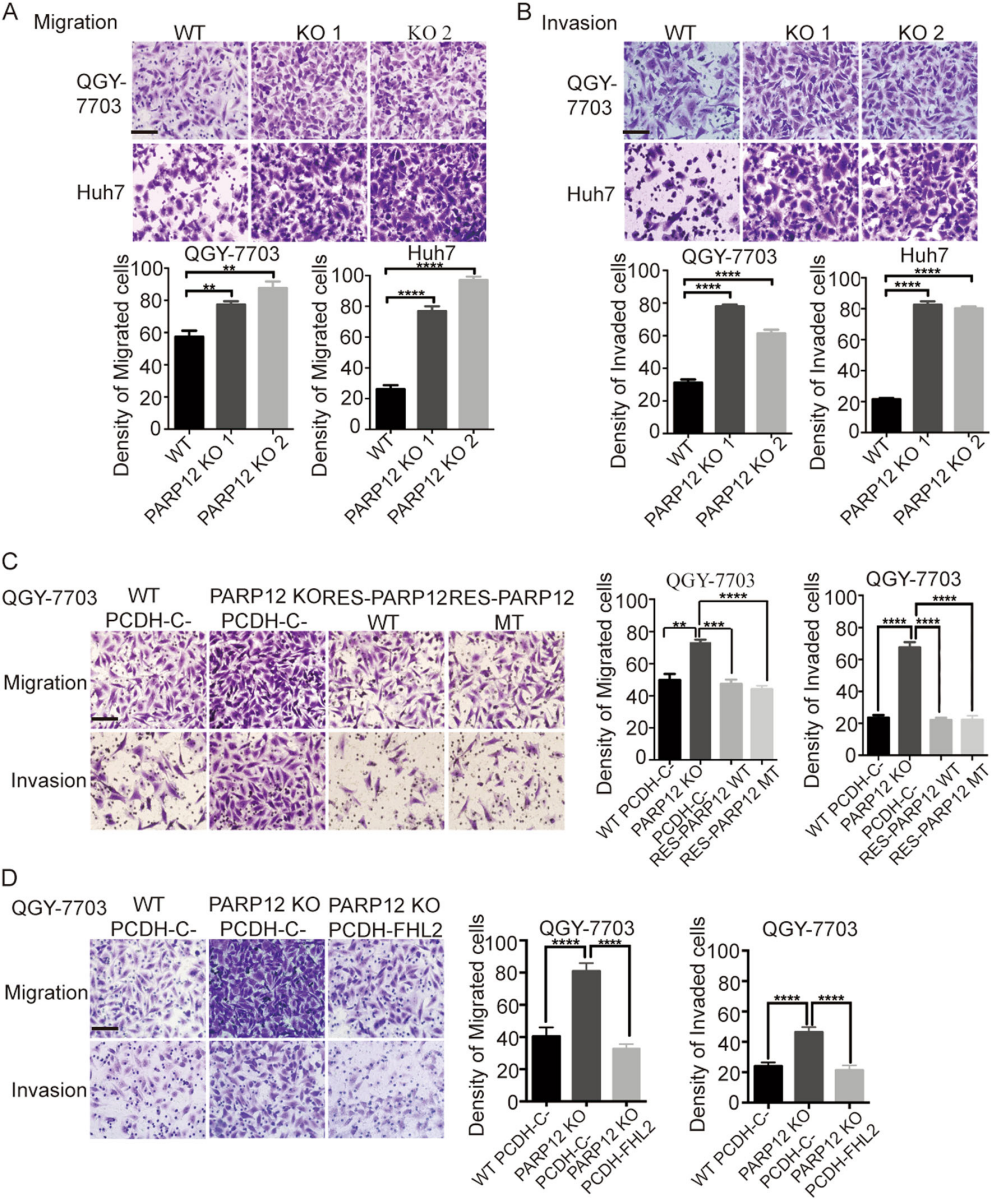
PARP12 regulates HCC cell migration and invasion. **a** PARP12 deficiency promotes QGY-7703 and Huh7 cell migration. Representative images of the effect of PARP12 deficiency on migrated cells are shown and originally magnified 400×. The histograms show the mean numbers of migrated cells from three independent tests (mean ± s.d.). **P < 0.01, ****P < 0.0001. Scale bars = 50 µm. **b** PARP12 deficiency promotes QGY-7703 and Huh7 cell invasion. Representative images of the effect of PARP12 deficiency on cell invasion are shown and originally magnified 400×. The histograms show the mean numbers of invasive cells from three independent tests (mean ± s.d.). ****P < 0.0001. Scale bars = 50 μm. **c** Wild-type and enzyme-inactive mutant PARP12 reconstitutions decreased the migration and invasion of PARP12-deficient QGY-7703 cells. Representative images of migrated and invaded cells are shown and originally magnified 400×. The histograms show the mean numbers of migrated and invaded cells from three independent tests (mean ± s.d.). **P < 0.01, ***P < 0.001, ****P < 0.0001. Scale bars = 50 µm. **d** FHL2 overexpression decreased the migration and invasion of PARP12-deficient QGY-7703 cells. Representative images of migrated and invaded cells are shown and originally magnified 400×. The histograms show the mean numbers of migrated and invaded cells from three independent tests (mean ± s.d.). ****P < 0.0001. Scale bars = 50 µm

EMT is an important process during cancer metastasis through which epithelial cells acquire mesenchymal properties and show reduced intercellular adhesion and increased motility^26,27^. EMT is involved in cell migration and invasion. As such, PARP12 may also regulate cell migration and invasion. To test this hypothesis, we examined the migration and invasion of wild-type or PARP12-deficient cells. In Fig. 6a, b, Transwell assay revealed that PARP12 deficiency dramatically promoted the migration and invasion of QGY-7703 and Huh7 cells, suggesting that PARP12 participated in HCC cell migration and invasion. Wild-type or enzyme-inactive mutant PARP12 reconstitution or FHL2 overexpression suppressed the migration and invasion of PARP12-deficient cells (Fig. 6c, d and Supplementary Figure 7). These results indicated that PARP12 regulated HCC cell migration and EMT through FHL2.

### PARP12 deficiency promotes HCC metastasis in vivo

**Fig. 7:**
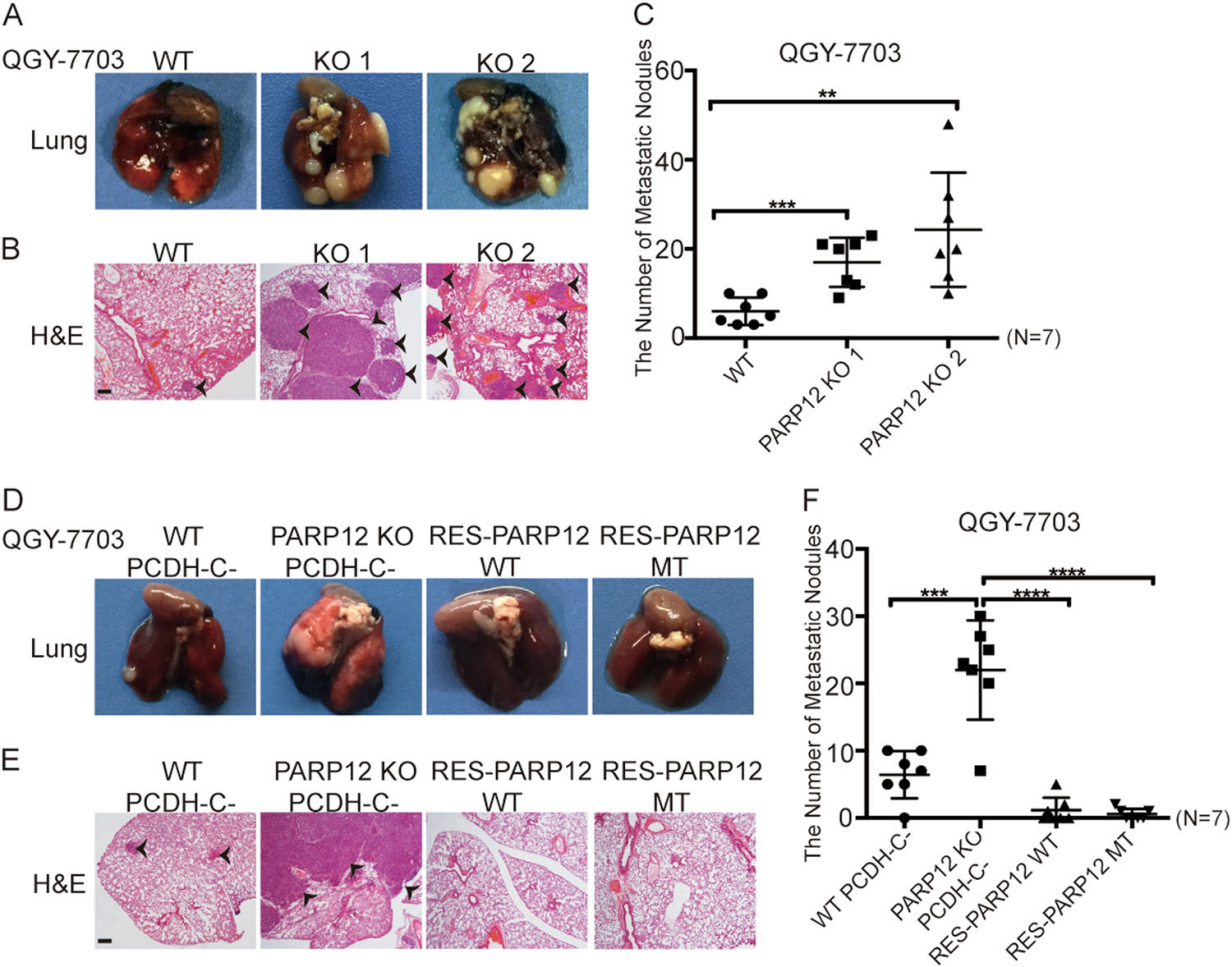
PARP12 deficiency promotes tumor metastasis in vivo. **a**–**c** PARP12 deficiency promotes tumor metastasis. PARP12 wild-type and -deficient QGY-7703 cells were injected into the nude mice through the tail vein. Representative images of metastatic tumors in the lung (**a**) and the H&E staining results, scale bars = 2.0 mm, (**b**) are shown. The scatter gram (**c**) shows the numbers of tumor nodules of each mouse (*n* = 7). **P < 0.01, ***P < 0.001. **d**–**f** Wild-type and enzyme-inactive mutant PARP12 reconstitutions decreased the tumor metastasis of PARP12-deficient QGY-7703 cells. PARP12 wild-type, -deficient, and -reconstituted QGY-7703 cells were injected into the nude mice through the tail vein. Representative images of metastatic tumors in the lung (**d**) and the H&E staining results, scale bars = 2.0 mm, (**e**) are shown. The scatter gram (**f**) shows the number of tumor nodules of each mouse (*n* = 7). ***P < 0.001, ****P < 0.0001

We examined the function of PARP12 on metastasis in vivo. Wild-type and PARP12-deficient QGY-7703 cells were injected into BALB/c nude mice through the tail vein. Six weeks after injection, the mice were killed, and the metastatic lung tumors were measured and analyzed. In Fig. 7a, b, the number and size of lung tumors significantly increased in the mice injected with PARP12-deficient QGY-7703 cells compared with those of the wild-type QGY-7703 cells, which formed few metastatic lung tumors in all seven injected mice, suggesting that PARP12 deficiency promoted tumor metastasis in vivo. Histological analysis indicated the presence of metastatic tumors in the lungs of these mice (Fig. 7c). Consistent with the migration and invasion results, the re-expression of the wild-type or enzyme-inactive mutant PARP12 could suppress the metastasis of PARP12-deficient cells in vivo (Fig. 7d–f). These results suggested that PARP12 suppressed HCC metastasis in vivo.

## Discussion

Metastases account for the great majority of cancer-associated deaths and remain the greatest challenge in the clinical management of cancer; however, this complex process remains the least understood aspect of cancer biology^28,29^. The EMT program is critical to the invasion and dissemination of most and possibly all carcinoma types and implicated in cancer cell invasion and migration^30^. The EMT program is activated by various signals, including TGF-βs, Wnts, and certain interleukins^31–33^. TGF-β is a cytokine that plays crucial roles in many biological processes^34,35^. During the early phase of cancer progression, TGF-β1 frequently acts as a tumor suppressor; in the later phase, TGF-β1 can become a tumor promotor^36,37^. TGF-β signaling promotes EMT by increasing the expression of mesenchymal markers and reducing the expression of epithelial markers in the late phase of cancer progression^38^. FHL2 is an important negative regulator of TGF-β1 transcription in the liver by both direct and indirect mechanisms^23^. Here, we found that PARP12 interacted with FHL2 and stabilized it by inhibiting its ubiquitination, suggesting that PARP12 might be a negative regulator of TGF-β1. We demonstrated that PARP12 deficiency increased the transcription of TGF-β1, promoted the EMT process, and supported the invasion and migration of HCC cells in vitro and in vivo, which are dependent on the protein level of FHL2. These results indicated that PARP12 was a tumor suppressor, especially in HCC metastasis.

Recently, we found that PARP10, another mono-ADP-ribosyltransferase, suppresses tumor metastasis dependent on the MARylation of Aurora A by PARP10^39^. In the present study, similar to wild-type PARP12, the inactive mutant PARP12 reconstitution could suppress the invasion and migration of PARP12-deficient cells, suggesting that PARP12 suppressed HCC metastasis independent of its enzymatic activity. These results also indicated that some mono-ADP-ribosyltransferases had important functions independent on its activity. Actually, it has been found that PARP1 suppresses the expression of interleukin 6 independent of its enzymatic activity^40^. These results implied that the functions of ADP-ribosyltransferases in tumor development are complicated, possibly limiting the use of PARP inhibitors in clinical treatments. Some PARPs negatively regulate tumor development, while other PARPs positively regulate tumor development. However, most PARP inhibitors suppress the enzymatic activity of poly-ADP-ribosyltransferases and mono-ADP-ribosyltransferases^3,41,42^. Special PARP inhibitors should be designed in the future. On the other hand, PARP inhibitors cannot suppress the functions of ADP-ribosyltransferases, which are independent on its enzymatic activity.

Overall, our findings revealed that mono-ADP-ribosyltransferase PARP12 plays an important role in the migration and invasion of HCC cells and the metastasis of HCC via the regulation of FHL2 stability and TGF-β1 expression.

## Materials and methods

### Cell culture and transfection

Human HCC cell lines (QGY-7703 and Huh7) and HEK293T cells were obtained from the Shanghai Cell Bank of Chinese Academy of Sciences (Shanghai, China) and American Type Culture Collection (ATCC, USA), respectively. All these cells were cultured in Dulbecco’s modified Eagle’s medium (DMEM) supplemented with 10% fetal bovine serum (FBS) and 1% penicillin and streptomycin at 37 °C in a humidified incubator with 5% CO_2_.

For transfection, HEK293T or HCC cells at 70% confluence were transfected with plasmids by using Lipofectamine® 2000 or Lipofectamine® 3000 (Invitrogen) in a serum-free medium according to the manufacturer’s instructions. After 6 h of transfection, the medium was replaced with a fresh complete medium.

### Plasmids

Human PARP12 (NCBI Accession NO.NP_073587.1) and FHL2 (NCBI Accession NO.NP_001034581.1) were cloned into modified pcDNA3.1 and pRIES2-EGFP vectors to generate constructs encoding (HA)-tagged PARP12 and FHL2 and S-protein/Flag/SBP (SFB) triple-tagged PARP12 and FHL2, respectively. The point mutants of PARP12 were generated by using a Quick Change site-directed mutagenesis kit (Stratagene) in accordance with the manufacturer’s protocol. PGEX-4T-1 and pET-28a vectors were used to generate GST- or His-tagged fusion proteins. sgRNAs that targeted PARP12 were designed via http://crispr.mit.edu website. sgRNA (5ʹ-TCTTGTTTCAGAACGACCCC-3ʹ) was subcloned into a pX335-U6-Chimeric-BB-CBh-hSpCas9n vector.

### Antibodies and reagents

Anti-Flag (F3165), anti-PARP12 (HPA003584), and anti-FHL2 (HPA006028) antibodies were purchased from Sigma. Anti-HA (ab18181) and anti-GAPDH (ab8245) antibodies were purchased from Abcam (Cambridge, MA, USA). Anti-snail (3879S), anti-N-cadhenrin (14215S), and anti-vimentin (5741S) antibodies were purchased from Cell Signaling Technology (Beverly, MA, USA). Anti-GST, anti-His, and anti-GFP antibodies were obtained from Abmart. Anti-(ADP-ribose) antibody (A01316) was purchased from GenScript

Glutathione Sepharose and Ni Sepharose were purchased from GE Heathcare (Little Chalfont, UK, EU). High-capacity Streptavidin Agarose (20359) and S-protein Agarose (69704) were bought from Thermo Scientific and Novagen, respectively.

### Generation of PARP12-deficient and PARP12-reconstituted cells

PARP12-deficient cells were generated using the CRISPR-Cas9 system. A constructed vector that targeted exon 2 of PARP12 was transiently transfected into QGY-7703 and Huh7 cells. After 48 h, the transfected cells were selected with puromycin for 2 days, and single colonies were screened through Western blot by using the anti-PARP12 antibody. The sgRNA-targeting genomic regions of positive clones were amplified and sequenced.

PARP12-reconstituted cells were established through lentivirus infection. In brief, wild-type or inactive mutant PARP12 or FHL2 lentivirus was packaged by the co-transfection of PCDH-PARP12 or PCDH-FHL2 with PLP1, PLP2, and VSVG into HEK293T cells. After 48 h, the cell culture medium was collected and mixed well with GML-PC^TM^ (Genomeditech) in accordance with the manufacturer’s protocols to obtain the packaged lentivirus. PARP12-deficient QGY-7703 and Huh7 cells were infected with the control or the indicated lentivirus and verified through Western blot by using the indicated antibodies.

### Affinity purification and identification of PARP12-associated proteins

Twenty dishes of HEK293T cells that stably expressed SFB-tagged PARP12 and SFB-tag (as a negative control) were collected and washed with phosphate-buffered saline (PBS). The cells were lysed with 5 ml ice-cold NETN buffer (20 mM Tris-HCl pH 8.0, 100 mM NaCl, 1 mM EDTA, and 0.5% Nonidet P-40) for 15 min on ice, and whole-cell lysates were obtained through centrifugation at 4 °C. The soluble fraction was incubated with 0.2 ml of high-capacity streptavidin beads at 4 °C for 2 h. The beads were washed three times with NETN buffer. The associated proteins were eluted with 2 mM biotin in 1× PBS and incubated with 50 µl of S beads at 4 °C for an additional 2 h. After five washes with NETN buffer, the pellets were boiled in SDS loading buffer (4% SDS, 20% glycerol, 10% 2-mercaptoethanol, 0.004% bromophenol blue, 0.125 M Tris-HCl) for 8 min and subjected to SDS-PAGE followed by Coomassie Brilliant Blue staining. Whole lanes were excised from the gel and analyzed through mass spectrometry in accordance with the standard protocol.

### Protein expression and purification

GST or His fusion proteins were expressed in *E. coli* BL21 (DE3) pLysS. In brief, the cells were grown in Luria–Bertani medium supplemented with 0.1 mg/ml kanamycin at 37 °C to OD_600_ of 0.6, and fusion proteins were induced by adding isopropyl β-d-1-thiogalactopyranoside (IPTG, 0.2 mM) for 12 h at 20 °C. The Cells were collected, resuspended in NETN buffer, and sonicated. The supernatant was collected and incubated with Glutathione Sepharose or Ni Sepharose for 3 h at 4 °C. After five washes with NETN buffer, the fusion proteins were eluted with the indicated elution buffer at 4 °C in accordance with the manufacturer’s protocols.

### Western blot

Proteins were separated through SDS-PAGE and transferred onto a polyvinylidene fluoride membrane (GE Healthcare) by using a semi-dry transfer unit (Bio-Rad). The membranes were blocked in TBST containing 5% non-fat milk and 0.1% Tween-20, incubated with the indicated primary antibodies for 2 h at room temperature, and incubated with HRP-conjugated secondary antibodies at room temperature for 1 h. Immunoreactivity was visualized with an ECL chemiluminescence system (Santa Cruz).

### ADP-ribosylation assay

For the in vitro ADP-ribosylation assays, 0.5 µg of recombinant His-PARP12 and 1 µg of recombinant GST-FHL2 were incubated with or without 25 µM biotin-NAD^+^ in 30 µl reaction buffer (50 mM Tris-HCl, pH 8.0, 1 mM DTT, 5 mM MgCl_2_, 50 nM DNA) for 30 min at 30 °C. The reactions were stopped by the addition of 2× SDS loading buffer, and the samples were analyzed through Western blot by using the indicated antibodies.

### Co-IP

The cells were lysed with NETN buffer (20 mM Tris-HCl at pH 8.0,100 mM NaCl, 1 mM EDTA, and 0.5% Nonidet P-40) for 10 min on ice, and whole-cell lysates were obtained through centrifugation at 4 °C. For exogenous IP, the lysates were incubated with high-capacity streptavidin beads for 3 h on a shaker at 4 °C. For endogenous IP, cell lysates were incubated with protein-A/G beads coupled with indicated antibodies for 3 h at 4 °C. After five washes with NETN buffer, the pellets were boiled in SDS loading buffer for 8 min, subjected to SDS-PAGE, and analyzed with the indicated antibodies.

### Pull-down assays

The cell lysates of HEK293T cells expressing GFP-tagged proteins were incubated with 3 mg of the His-tagged macro domain of PARP14 immobilized on Ni Sepharose (GE Healthcare) for 3 h at 4 °C. After five washes with 1× NETN lysis buffer, the pellets were boiled in SDS loading buffer for 8 min, subjected to SDS-PAGE, and analyzed with the indicated antibodies.

### Ubiquitination assay

For in vivo ubiquitination, SFB-FHL2 and HA-Ub were co-transfected into PARP12 wild-type or deficient cells. After 48 h, the cells were collected and lysed with 1× NETN cell lysis buffer. Flag-FHL2 was immunoprecipitated with streptavidin beads and analyzed with Western blot by using the indicated antibodies.

### CHX treatment

For the protein half-life assay, PARP12 wild-type, deficient, and reconstituted cells were treated with 100 µg/ml CHX and harvested at the indicated time points. The cells were lysed in the SDS loading buffer, boiled for 8 min at 98 °C, and analyzed through Western blot by using the indicated antibodies.

### Quantitative real-time PCR

Total RNA was extracted from QGY-7703 and Huh7 cells by using TRIzol reagent (Life Technology) in accordance with the manufacturer’s instructions. Reverse transcription PCR was performed by using a RT-PCR kit (Toyobo). Real-time quantitative PCR was conducted with SYBR Green Master Mix (Toyobo). The cycling parameters were 98 °C for 20 s, 58 °C for 20 s, and 72 °C for 20 s for 40 cycles. Melting curve analysis was performed, and relative expression levels were determined using the 2^-ΔΔCt^ method. Primer pairs used were as follows:

**Table:** 

Gene	RT-qPCR primers
β_2_-MG	F:5ʹ-ATGAGTATGCCTGCCGTGTGAAC-3ʹ R:5ʹ-TGTGGAGCAACCTGCTCAGATAC-3ʹ
FHL2	F:5ʹ-AGAGTTTCATCCCCAAAGACAA-3ʹ R:5ʹ-AGTTCAGGCAGTAGGCAAAGTC-3ʹ
N-cadherin	F:5ʹ-CGCCATCCAGACCGACCCAA-3ʹ R:5ʹ-GTCGATTGGTTTGACCACGGTGAC-3ʹ
E-cadherin	F:5ʹ-CACCCTGGCTTTGACGCCGA-3ʹ R:5ʹ-AAAATTCACTCTGCCCAGGACGCG-3ʹ
Vimentin	F:5ʹ-GACGCCATCAACACCGAGTT-3ʹ R:5ʹ-CTTTGTCGTTGGTTAGCTGGT-3ʹ
Snail	F:5ʹ-TCGGAAGCCTAACTACAGCGA-3ʹ R:5ʹ-AGATGAGCATTGGCAGCGAG-3ʹ
TGF-β1	F:5ʹ-GCCTTTCCTGCTTCTCATGG-3ʹ R:5ʹ-TCCTTGCGGAAGTCAATGTAC-3ʹ

### Migration and invasion assays

A total of 2 × 10^4^ QGY-7703 cells or Huh7 cells in 100 µl of serum-free medium were seeded into the upper chamber, and 800 µl of serum-containing medium was added to the lower chamber of 24-well Transwell plates (Corning). After 24 h of culturing, the cells were fixed in 4% paraformaldehyde in PBS for 30 min and stained with a crystal violet solution for 1 h. Five randomly chosen visual fields were recorded under a microscope and analyzed statistically with ImageJ (NIH). For invasion assays, the upper chamber was pre-coated with Matrigel (BD Biosciences), and 2 × 10^4^ QGY-7703 cells or Huh7 cells were seeded onto the upper chamber.

### In vivo metastasis assay

Male nude mice were purchased from the Institute of Materia Medica (CAS, Shanghai, China) and cared for in accordance with the National Institutes of Health Guide for the Care and Use of Laboratory Animals. All the experimental protocols were approved in advance by the Ethics Review Committee for the Animal Experimentation of Fudan University. Afterward, 1.0 × 10^6^ cells were suspended in 200 μl of PBS and injected into the nude mice through the tail vein. After 8 weeks, the mice were sacrificed to examine the number of metastatic tumors in their lungs.

### ELISA assay

A total of 1 × 10^5^ cells were seeded into 12-well plates and cultured with DMEM without FBS. After 48 h, the cell culture media were collected to analyze TGF-β1 level by using a Human TGF-β1 ELISA kit (Proteintech) in accordance with the manufacturer’s protocols.

### Statistical analysis

All the experiments were repeated at least thrice. The samples or animals were allocated to experimental groups through a random assignment. Data were analyzed through a two-tailed Student’s *t*-test. Statistical significance levels were set at *P *<* 0.05, **P < 0.01, and ***P < 0.001. Biostatistical analysis was carried out using GraphPad (GraphPad Prism 5, Lajoua, California, USA).

## Electronic supplementary material

SUPPLEMENTAL MATERIAL
