# Supplementary material for: PARP12 (ARTD12) suppresses hepatocellular carcinoma metastasis through interacting with FHL2 and regulating its stability
Source: Cell Death Dis. 2018 Aug 28;9(9):856. doi: 10.1038/s41419-018-0906-1 (PMC6113207; doi:10.1038/s41419-018-0906-1)

**Supplementary Figure 1. PARP12 interacts with FHL2 in HCC cell lines.**

(A, B) Endogenous PARP12 interacts with FHL2 in QGY-7703 and Huh7 cell lines. Lysates from QGY-7703 and Huh7 cells were subjected to immunoprecipitation and western blot with the indicated antibodies respectively. An irrelevant IgG was used as the negative control.

**Supplementary Figure 2. PARP12-deficient QGY-7703 and Huh7 cells were generated by CRISPR-Cas9 system.**

(A) sgRNA targeting to PARP12 exon 2 was designed according to the website <http://crispr.mit.edu>. (B) PARP12-deficient QGY-7703 and Huh7 cell lines were identified by DNA sequencing. The genomic region of PARP12 targeted by CRISPR-Cas9 was amplified by PCR and subjected to DNA sequencing.

**Supplementary Figure 3. PARP12 regulates the protein stability of FHL2 in Huh7 cells.**

(A) Wild type and enzyme inactive mutant PARP12-reconstitutions increased the protein level of FHL2 in PARP12- deficient Huh7 cells. The protein levels of FHL2 in PARP12 wild type, deficient and reconstituted Huh7 cells were examined by western blot using anti-FHL2 antibody, where β-tubulin was used as a loading control. (B) Wild type and enzyme inactive mutant PARP12-reconstitutions stabilized FHL2 in PARP12-deficient Huh7 cells. Wild type or PARP12-deficient and reconstituted Huh7 cells were treated with 100 μg/ml cycloheximide (CHX), collected at indicated time points and then immunoblotted with antibodies against FHL2 and β-tubulin.

**Supplementary Figure 4. PARP12 regulates the interaction between FHL2 and TRAF6**

(A, B) PARP12 defeciency enhanced the interaction between FHL2 and TRAF6. HA-FHL2 and SFB-TRAF6 were co-transfected into wild type or PARP12 deficient QGY-7703 (A) and Huh7 (B) cells and applied to immunoprecipitation (IP), respectively. Whole-cell lysates were blotted and shown as input and detected with the indicated antibodies.

**Supplementary Figure 5. PARP12 negatively regulates TGF-β1 expression in Huh7 cells**

(A) Wild type and enzyme inactive mutant PARP12-reconstitutions decreased the transcription of TGF-β1 in PARP12-deficient Huh7 cells. The mRNA level of TGF-β1 in PARP12 wild type, deficient and reconstituted Huh7 cells were analyzed by RT-qPCR. “**”: P<0.01. (B) Wild type and enzyme inactive mutant PARP12-reconstitutions decreased the TGF-β1 level in PARP12-deficient cell culture medium. The TGF-β1 levels in PARP12 wild type, deficient and reconstituted Huh7 cell culture medium were analyzed by ELISA. “***”: P<0.001.

**Supplementary Figure 6. PARP12 regulates EMT process in Huh7 cells.**

(A) The mRNA levels of CDH1, CDH2, Vimentin and Snail were examined in PARP12 wide type, deficient and PARP12-reconstitution Huh7 cells by RT-qPCR. “*”: P<0.05, “***”: P<0.001, “****”: P<0.0001. (B) The protein levels of N-Cadherin, Vimentin and Snail in PARP12 wide type, deficient and reconstituted Huh7 cells were examined by western blot using indicated antibodies, where β-tubulin was used as a loading control.

**Supplementary Figure 7. PARP12 regulates migration and invasion of Huh7 cells.**

(A)Wild type and enzyme inactive mutant PARP12-reconstitutions decreased the migration and invasion of PARP12-deficient Huh7 cells. Representative images of migrated and invaded cells were shown and originally magnified 400×. Scale bars=50μm. (B)The histograms show the mean numbers of migrated and invaded cells from three independent tests (mean±s.d.). “****”: P<0.0001.


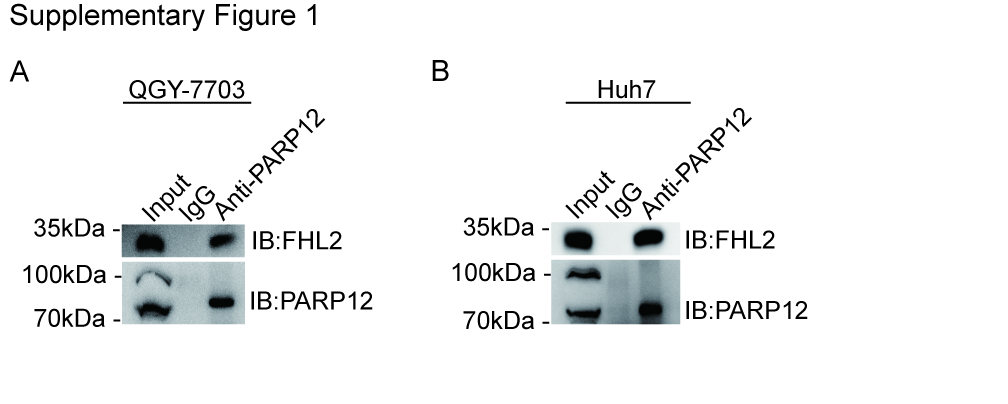


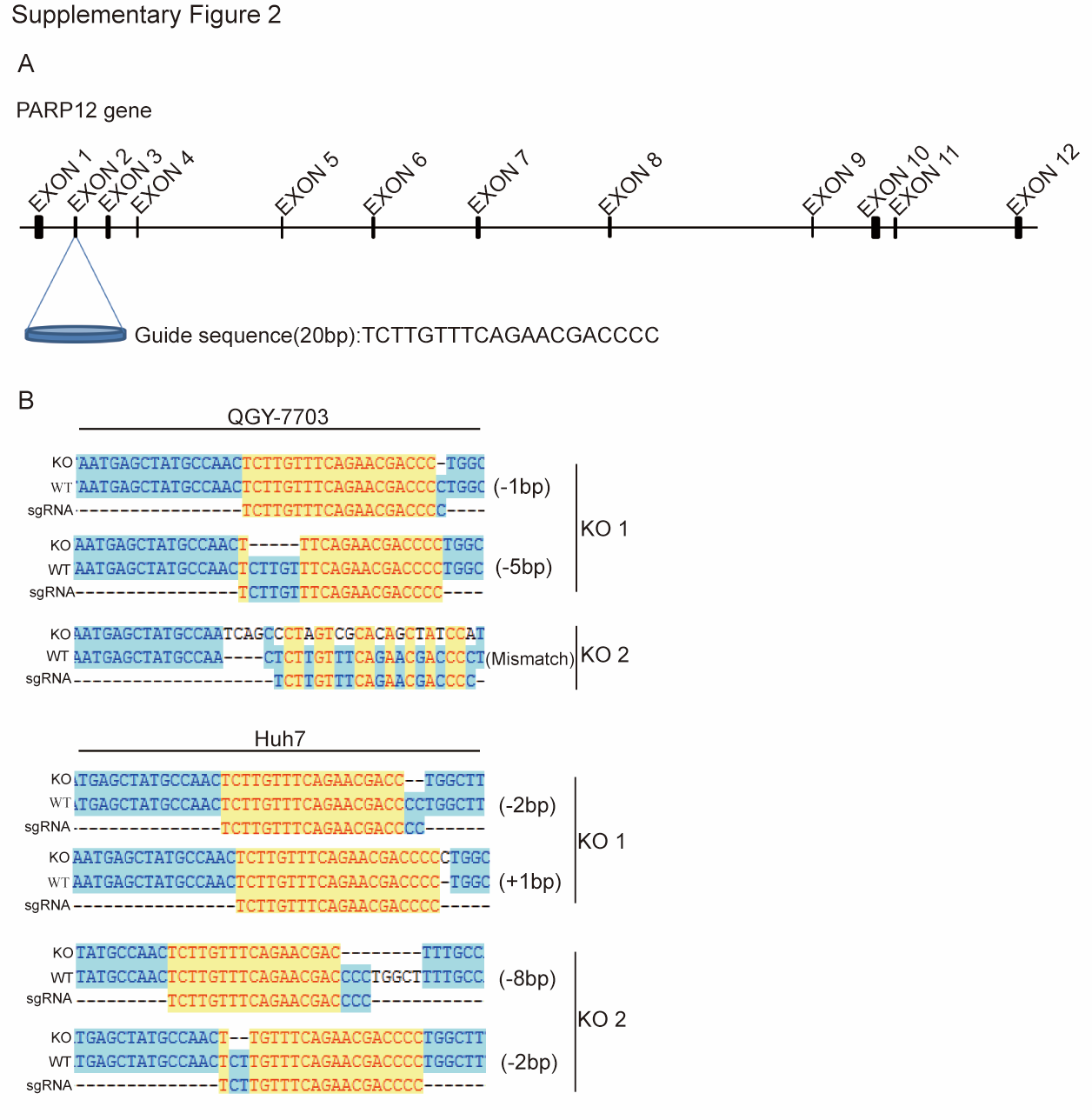


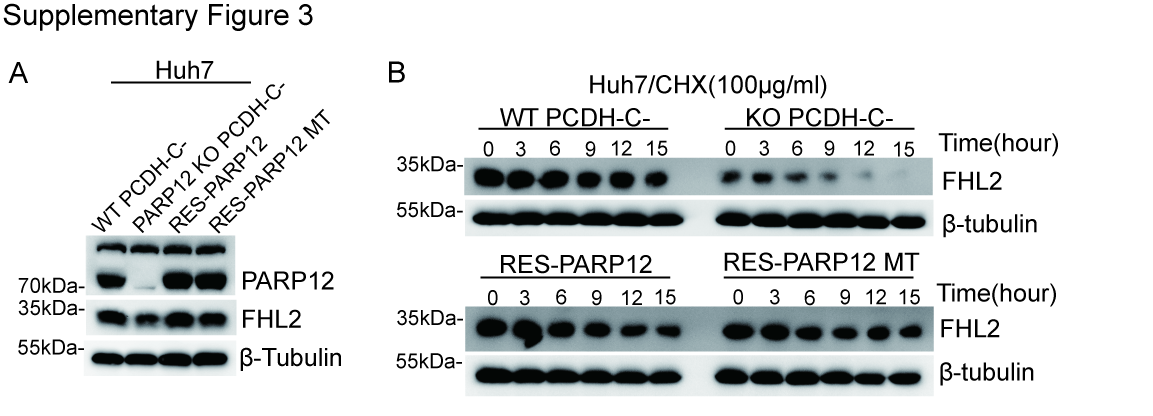


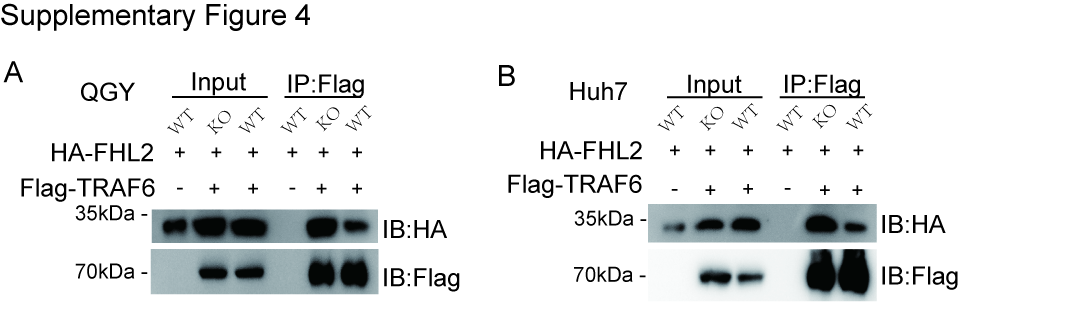


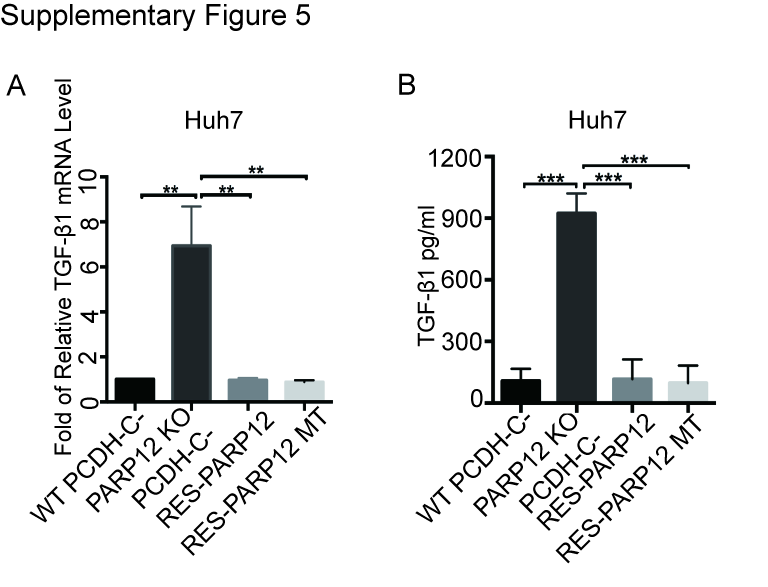


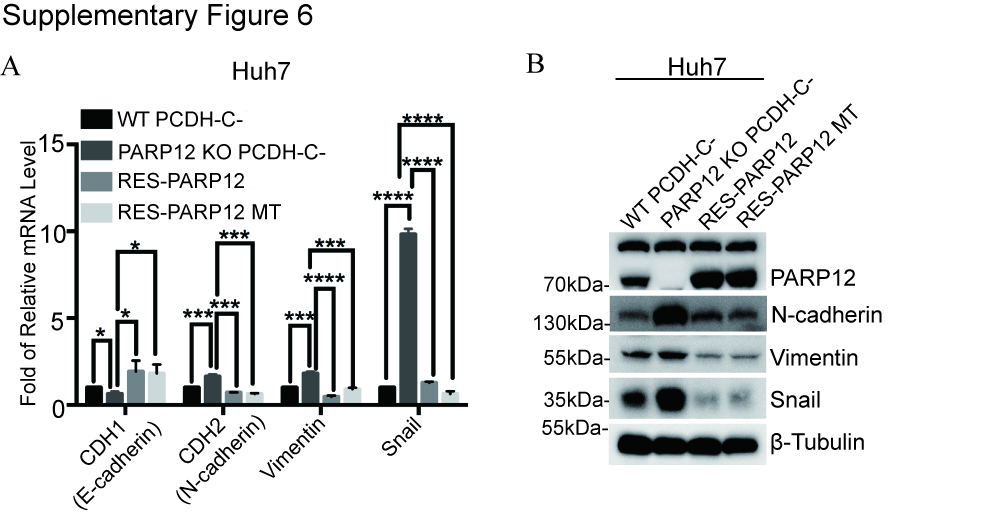


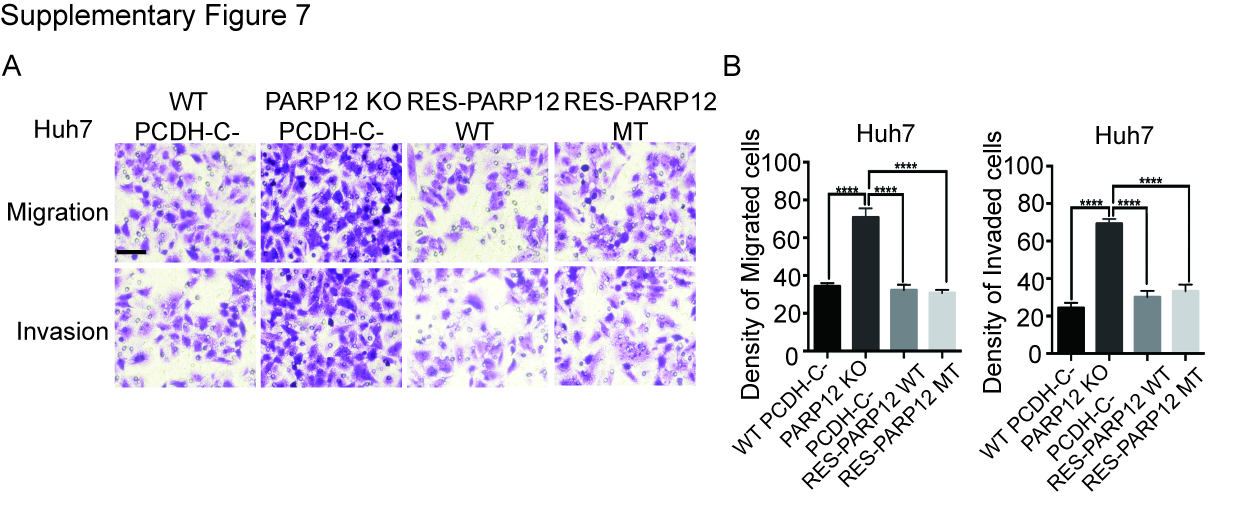

Supplement: Supplementary file 1 — SUPPLEMENTAL MATERIAL [file 41419_2018_906_MOESM1_ESM.docx]
